# Inorganic Nitrogen Leaching from Organic and Conventional Rice Production on a Newly Claimed Calciustoll in Central Asia

**DOI:** 10.1371/journal.pone.0098138

**Published:** 2014-05-23

**Authors:** Fanqiao Meng, Jørgen E. Olesen, Xiangping Sun, Wenliang Wu

**Affiliations:** 1 College of Resources and Environmental Sciences, China Agricultural University, Beijing, China; 2 Department of Agroecology and Environment, Faculty of Agricultural Sciences, Aarhus University, Tjele, Denmark; DOE Pacific Northwest National Laboratory, United States of America

## Abstract

Characterizing the dynamics of nitrogen (N) leaching from organic and conventional paddy fields is necessary to optimize fertilization and to evaluate the impact of these contrasting farming systems on water bodies. We assessed N leaching in organic versus conventional rice production systems of the Ili River Valley, a representative aquatic ecosystem of Central Asia. The N leaching and overall performance of these systems were measured during 2009, using a randomized block experiment with five treatments. PVC pipes were installed at soil depths of 50 and 180 cm to collect percolation water from flooded organic and conventional paddies, and inorganic N (NH_4_-N+NO_3_-N) was analyzed. Two high-concentration peaks of NH_4_-N were observed in all treatments: one during early tillering and a second during flowering. A third peak at the mid-tillering stage was observed only under conventional fertilization. NO_3_-N concentrations were highest at transplant and then declined until harvest. At the 50 cm soil depth, NO_3_-N concentration was 21–42% higher than NH_4_-N in percolation water from organic paddies, while NH_4_-N and NO_3_-N concentrations were similar for the conventional and control treatments. At the depth of 180 cm, NH_4_-N and NO_3_-N were the predominant inorganic N for organic and conventional paddies, respectively. Inorganic N concentrations decreased with soil depth, but this attenuation was more marked in organic than in conventional paddies. Conventional paddies leached a higher percentage of applied N (0.78%) than did organic treatments (0.32–0.60%), but the two farming systems leached a similar amount of inorganic N per unit yield (0.21–0.34 kg N Mg^−1^ rice grains). Conventional production showed higher N utilization efficiency compared to fertilized organic treatments. These results suggest that organic rice production in the Ili River Valley is unlikely to reduce inorganic N leaching, if high crop yields similar to conventional rice production are to be maintained.

## Introduction

Nitrogen (N) leaching is one of the primary pathways of N loss from flooded paddy farmland, representing 30–50% of total N loss from such soils [1]. The amount of leached N may equal up to 15% of the total applied N in rice (*Oryza sativa* L.) production [2], [3]. Flooded rice crops typically capture only 20–40% of N applied in the harvested grain [4]. N utilization efficiency can be improved by reducing the various forms of loss, including leaching. Because reactive N is highly soluble, its subsequently high mobility in the environment makes it a major contributor to surface and groundwater contamination. Ju et al. [5] reported that high inputs (550–600 kg N ha^−1^) associated with current rice production systems in China did not significantly increase crop yields, yet cause a doubling of N losses to the environment, mainly from increased denitrification in waterlogged systems. Accordingly, understanding N loss processes, particularly through leaching and N_2_O emission, is necessary to both improve resource use efficiency and to protect the quality of nearby water bodies. In this study, we focused on monitoring N leaching losses at different rice growth stages and soil depths in organic and conventional rice production systems.

Located in the Xinjiang Uygur Autonomous Region of northwestern China, the Ili River Valley has become an important grain production region, owing to its plentiful surface water, well-developed soils, and extensive meadow grasslands. Of the total land area (5.82 million ha), approximately 1 million ha are under cultivation, and the introduction of new irrigation technologies is facilitating the conversion of meadow grasslands to agricultural uses [6]. However, the Ili River is sensitive to pollution, which is of local and international concern as the river flows into Balkhash Lake in Kazakhstan. The river receives pollutants from both surface run-off and drainage from farming activities throughout the valley. In addition to water quality concerns, the terrestrial ecosystem may also be at risk because it contains shallow soils with a coarse texture. As such, organic farming is being promoted for the conservation of soil and water resources to reduce the overall negative environmental impact of agricultural production, as well as to increase farmer's income. However, it is unclear whether organic production can maintain high rice yields, with or without high fertilizer inputs, and what effect shifting from conventional to organic farming may have on N loading to the Ili River.

Many studies have examined N leaching from organic and conventional production [7], [8]. Such research has revealed that organic farming may lower N leaching compared to conventional systems, both by reducing N inputs and by including catch crops (cover crops) in the rotation [8], [9], although the magnitude of this effect is subject to high uncertainty due to variation in crop yields and input intensities [9], [10]. Although rice dominates grain production in many developing countries including China, little research has been conducted in this Central Asia region about N loss in organic versus conventional rice farming, especially regarding N loss through percolation or leaching [2], [4], [11].

This study compared the inorganic N (NH_4_-N and NO_3_-N) in percolation/leachate water from organic versus conventional production systems with high yields of rice production, by the application of high-loads of animal manure. We hypothesized that organic rice farming leaches less inorganic N compared with conventional rice in the Ili River Valley. In order to calculate the amount of N leached from paddies, we had to address the technical challenge of quantifying the total volume of leachate from a single-cropping rice paddy. To do so, we designed a filtration system using PVC pipes at two depths (50 and 180 cm) to capture the leachate, and quantified the percolation rates throughout the rice growing season using a water balance based on the difference between incoming (*i.e.*, irrigation and precipitation) and outgoing (evapotranspiration and surface runoff) flows. To better understand the potential environmental effects of the treatments, we evaluated N leaching not only in absolute terms, but also in terms of leachate per unit yield of rice and as a percentage of the total N applied.

## Materials and Methods

### Experiment site

**Figure 1:**
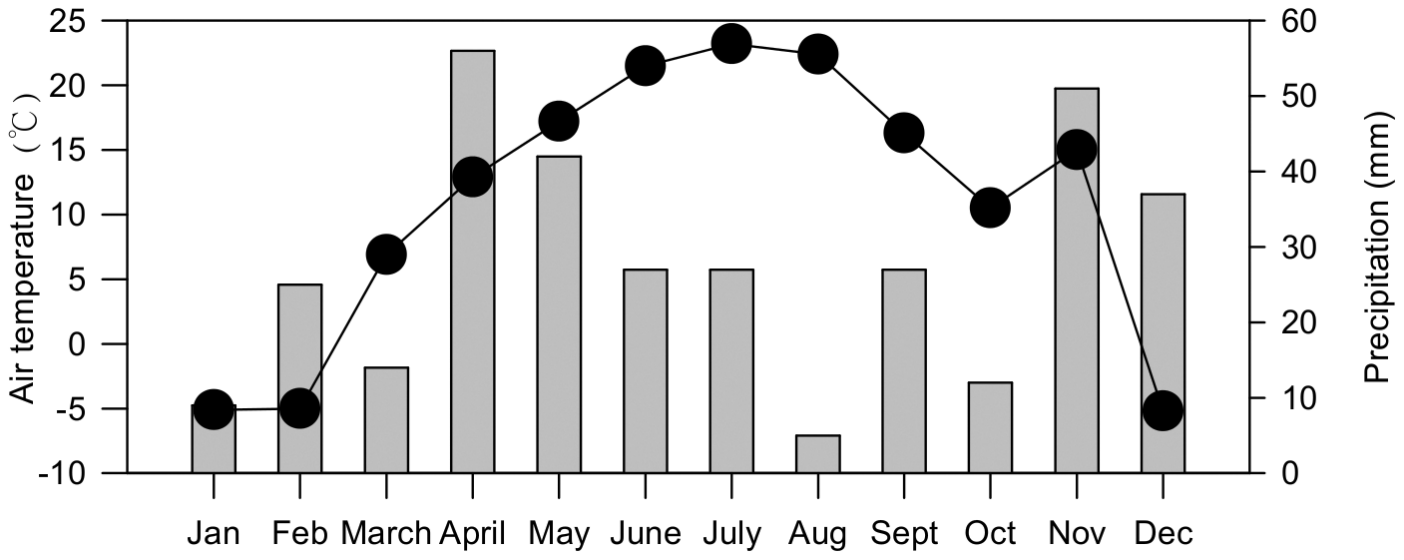
Air temperature and precipitation at the experimental site in Chabuchaer County for 2009.

The field experiment was conducted at Chabuchaer Farm (48°65'N, 80°06'E, 634 m ASL) in the Ili River Valley of the Xinjiang Uygur Autonomous Region, China. The region has a temperate continental climate with a mean annual temperature of 9.5°C and annual precipitation of 260 mm, which falls predominantly in June, July, and August. In 2009 when monitoring was implemented, precipitation mainly happened in April to July, November and December (Fig. 1). The farm is located on an alluvial plain off the south bank of the Ili River. The land use was converted to agriculture by the local government in 2005 as part of a national grain supply project. Because this land was newly reclaimed, we could examine the contrasting effects of conventional and organic farming techniques without the usual conversion period from conventional to organic farming, which can confound the results. Our study was initiated in 2008 and the data presented here were collected in the 2009 season.

The soil was a calciustoll with salt content of 1.4%. Rice had been continuously cultivated on this land since 2005, with the aim of reducing soil salinity. The soil is a sandy loam, with 3.2% clay, 44.7% silt, and 52.1% sand. Other important characteristics of the soil (0–20 cm) are as follows: 14.0 g kg^−1^ soil organic matter, 1.15 g kg^−1^ total N, 11 mg kg^−1^ Olsen phosphorous, 264 mg kg^−1^ available potassium, and a pH of 7.9.

Permission to conduct the experiment and sampling at the site was obtained from the Ili Agricultural Technical Extension Station, and the Farmer Liu Ermao. These field studies did not involve endangered or protected species.

### Crop management

Rice (*Oryza sativa* L., cultivar Nonglin-315) was transplanted to the experimental farm on May 29, 2009. The fertilizer used was either a conventional combination of mineral fertilizers with animal manure, as used in the local production system, or only organic fertilizers (composted animal manure [B] with castor (*Ricinuscommunis* L.) bean meal [A]), as used typically in organic rice production. The level of organic fertilization was manipulated to modify the overall N input. Hence, the five treatments were 1) an unfertilized control treatment (CK), 2) low-level organic fertilization (B1A1): composted animal manure at 15,000 kg ha^−1^ and castor bean meal at 2,250 kg ha^−1^, 3) mid-level organic fertilization (B2A1): composted animal manure at 45,000 kg ha^−1^ and castor bean meal at 2,250 kg ha^−1^, 4) high-level organic fertilization (B3A1): composted animal manure at 75,000 kg ha^−1^ and castor bean meal at 2,250 kg ha^−1^, and 5) conventional production system (LS): composted animal manure at 11,250 kg ha^−1^, urea at 277.5 kg ha^−1^, diammonium phosphate at 247.5 kg ha^−1^, and compounded mineral fertilizer at 75 kg ha^−1^ (Table 1). The treatments were arranged in a randomized block design with three replicates.

**Table 1:** Experimental treatments in terms of total nutrient inputs for N, P, and K (kg ha^−1^).

Treatment	Fertilization	Nutrient input (kg ha^−1^)		
		N	P	K
CK	Control with no fertilization	0	0	0
LS	Local conventional system: composted animal manure (27.6% of water content) at 11,250 kg ha^−1^, urea at 277.5 kg ha^−1^, diammonium phosphate at 247.5 kg ha^−1^, and compounded fertilizer at 75 kg ha^−1^.	297	83	117
B1A1	Composted animal manure (27.6% of water content) at 15,000 kg ha^−1^ plus castor bean meal (22.5% of water content) at 2,250 kg ha^−1^	193	52	163
B2A1	Composted animal manure (27.6% of water content) at 45,000 kg ha^−1^ plus castor bean meal (22.5% of water content) at 2,250 kg ha^−1^	498	125	446
B3A1	Composted animal manure (27.6% of water content) at 75,000 kg ha^−1^ plus castor bean meal (22.5% of water content) at 2,250 kg ha^−1^	802	199	728

Composted animal manure was applied on May 9 as the basal fertilizer for all treatments (except CK), 20 days before transplanting. The total amount of castor bean meal was halved and applied in two stages, first as topdressing at the early tillering stage (June 27) and then at the late tillering stage (July 24). Topdressings for LS were applied on June 27 (120 kg ha^−1^ diammonium phosphate with 75 kg ha^−1^ compounded fertilizer), July 6 (127.5 kg ha^−1^ diammonium phosphate with 127.5 kg ha^−1^ urea), and July 15 (urea, 150 kg ha^−1^). The nutrient contents of the fertilizers used in the experiments are listed in Table 2.

**Table 2:** Nutrient contents of fertilizers used in the experiments (% dry weight).

	*N*	*P*	*K*
Composted animal manure	1.40	0.34	1.30
Castor bean meal	2.37	0.84	1.10
Urea	46		
Diammonium phosphate	18	20	
Compounded fertilizer	14	6.98	12.45

Each paddy block had an area of 8×10 m = 80 m^2^. Flood irrigation (about 20 cm of standing water), popular in other Central Asian countries [4], was used continuously from June 1 to September 7, except during the first week after fertilization application. Hand weeding was used in all treatments, along with the addition of herbicides to conventional replicates. The main development stages for the rice were transplant (May 29-Jun 13), tillering and elongation (∼Aug 5 for organic paddies / ∼Aug 10 for conventional paddies), heading (Aug 5-Sep 1 for organic / Aug 10-Sep 5 for conventional), and ripening (Sep 1-Oct 2 for organic / Sep 5-Oct 13 for conventional).

### Measurements of N cycling

**Figure 2:**
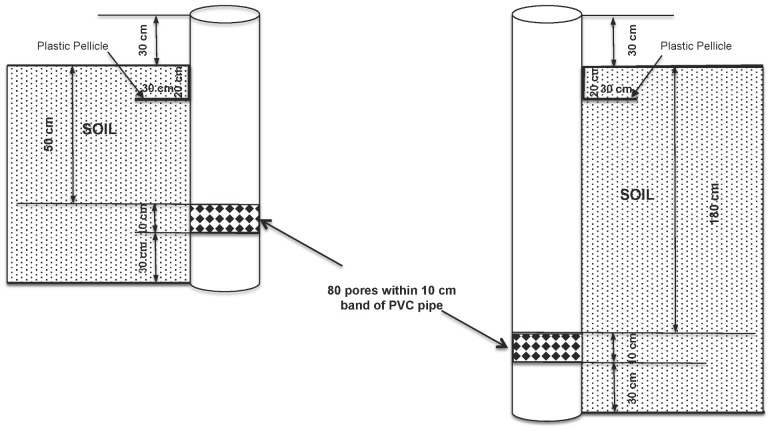
Schematic of standpipes for measuring percolate inorganic N concentrations in the paddy fields. Shaded area indicates the depth below the soil surface. Horizontal lines at 20-radium around the pipes.

Previous studies have shown that inorganic N (NH_4_-N+NO_3_-N), rather than particulate N, makes up 50–90% of the total N leached from the soils, and for organic fertilizers or fertile soil, this proportion maybe even higher [12], [13], [14]. Only inorganic N was analyzed in percolation water collected in our experiment. Percolation water was collected from flooded paddies using polyvinyl chloride (PVC) standpipes [14], [15], [16]. Two PVC pipes (5 cm in diameter, 150 and 250 cm in length) with sealed bottoms were installed roughly in the center of each field plot to collect drainage water from the saturated soil (Fig. 2). Both pipes were perforated 80 times (6-mm internal diameter) within a 10-cm-wide band, 30 cm from the bottom of the pipe. The porous zone of the pipe was wrapped with nylon textile to prevent sand in-filling. As the average soil layer is 2 m deep in this region, we compared the inorganic N leaching in 50 and 180 cm. At the later depth we considered that inorganic N was not usable by the rice. Thus, the pipes were installed at depths of 50 and 180 cm from the surface to the uppermost pore (Fig. 2). The gap between the collection pipe and soil wall in the porous zone was filled with quartz powder to prevent anything except soil solution from entering the pipe. At a depth of 20 cm, plastic film was wrapped around the PVC pipe, extending horizontally in a 30-cm radius from the pipe to reduce the preferential flow from the irrigation water.

Sampling was conducted 1 and 3 days after fertilization, and thereafter at intervals of 5 days to 2 weeks, depending on the percolate volume. Water samples were stored in plastic vials and refrigerated until analysis. Percolation and irrigation water were analyzed for inorganic N (NH_4_-N+NO_3_-N) content using a continuous flow N analyzer (TRAACS 2000). During the irrigation season, average NH_4_-N and NO_3_-N concentrations in the irrigation water were 0.11 and 0.68 mg L^−1^ respectively; these values were subtracted from the respective inorganic N concentrations in percolation water to calculate net N (i.e., the amount leached).

The volume of drainage water was calculated per unit area of paddy field. We considered the spatial range of percolation into the pipe to be a half-ellipsoid, having a width of 5 cm and a depth of either 50 or 180 cm, with diameters of 27.5 cm (50/2+5/2) or 92.5 cm (180/2+5/2) [15]. The volume of each ellipsoid was calculated as 4/3*π*ab ^2^, where *a* and *b* are the length of the long axis and radius of the ellipsoid, respectively. Each hectare of paddy field included 252,671 or 222,332 half-ellipsoids, at depths of 50 cm and 180 cm, respectively. Thus, total N leached per ha could be calculated by summing these half ellipsoids at each soil depth, as well as across time to calculate total N lost over one growth cycle. In total, percolation water was sampled 15 times from June 1 to September 7, the full growing season of the rice paddies.

The measured volume of percolation water in the paddy field was calibrated using the following equation [17]: 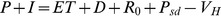
(1)Where *P* is the rainfall (mm) during the rice growth season; *I* is irrigation (mm); *ET* is evapotranspiration (mm), calculated using a lysimeter installed in the paddy plot; *D* is drainage through drain pipe (mm), excluding surface runoff and percolation; *R_o_* is surface runoff (mm); *P_sd_* is percolation (mm); and *V_H_* is the change of the water table in the paddy field (mm). Briefly, an iron lysimeter (1 m wide×1 m long×1.2 m high), filled with original paddy soil, was installed in the paddy field. The bottom of the lysimeter was perforated to allow percolated water to drip into a storage tank (0.5 m wide×0.5 m long×0.5 m high) welded to the lysimeter for storage of the percolated water. A meter stick was also fixed inside the lysimeter to measure the water table. Evapotranspiration from the paddy field was calculated according to Equation (1). In our study, there was no artificial drainage or surface runoff, so *D* and *R_o_* were set to 0. All of the terms are expressed in millimeters and then converted into m^3^ ha^−1^. One growth cycle (June 1 to September 7, 2009) was used to bound all calculations. The difference between *P_sd_* calculated from Equation (1) and from the measured percolation water volume was less than 10%; hence, here we presented percolated inorganic N as defined by its concentration (NH_4_-N and NO_3_-N) multiplied by measured percolate volume.

Relative N leaching loss (% of applied N) for a given plot was determined after first subtracting N leached from the nil treatment (CK) and then expressed as a percentage of the total N applied. Similarly, the environmental performance of organic and conventional fertilizer treatments, i.e., N leaching, was also evaluated based on the amount of inorganic N leached during the production of 1 Mg of rice (kg N Mg^−1^ grain). N utilization efficiency (NUE) was estimated using the equation (2): 
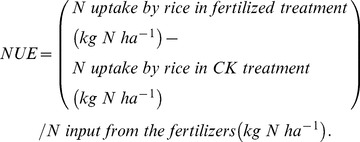
(2)


### Sampling and measurement of fertilizers, soils and plants

Manures were sampled to determine the content of dry matter (DM) and total N before spreading. Plant samples were taken in early October to obtain rice grain with husk and straw, by cutting stems at 2 cm above the ground from two 2 m^2^ areas per plot. The plant samples were washed with distilled water, oven-dried at 65°C, and weighed to determine rice biomass. Plant samples were then digested using H_2_SO_4_-H_2_O_2_, after which N, P, and K in the digestion solution were determined using the Kjeldahl method, calorimetrically by a Techniconautoanalyzer, and via atomic emission spectrometry (AES), respectively.

Statistical analyses

Cumulative inorganic N leached, net inorganic N leached per Mg of rice produced, and the proportion of net leached inorganic N in total N input were compared among different treatments using the least-significant difference test after a one-way analysis of variance for a randomized block design at the 0.05 significance level. The PROC MIXED procedure in SAS v.9.1 (SAS Institute, Cary, NC, USA) was performed to analyze the effects of fertilization on NH_4_-N and NO_3_-N concentrations over the sampling period (15 samplings), with the treatment as the fixed effect and sampling time as the random factor. Differences among means were calculated using a Differences of Least Squares Means with the PDIFF option and a Bonferroni adjustment method. The significance level was also at 0.05.

## Results

### Inorganic N concentrations in percolation water at 50 cm depth

**Figure 3:**
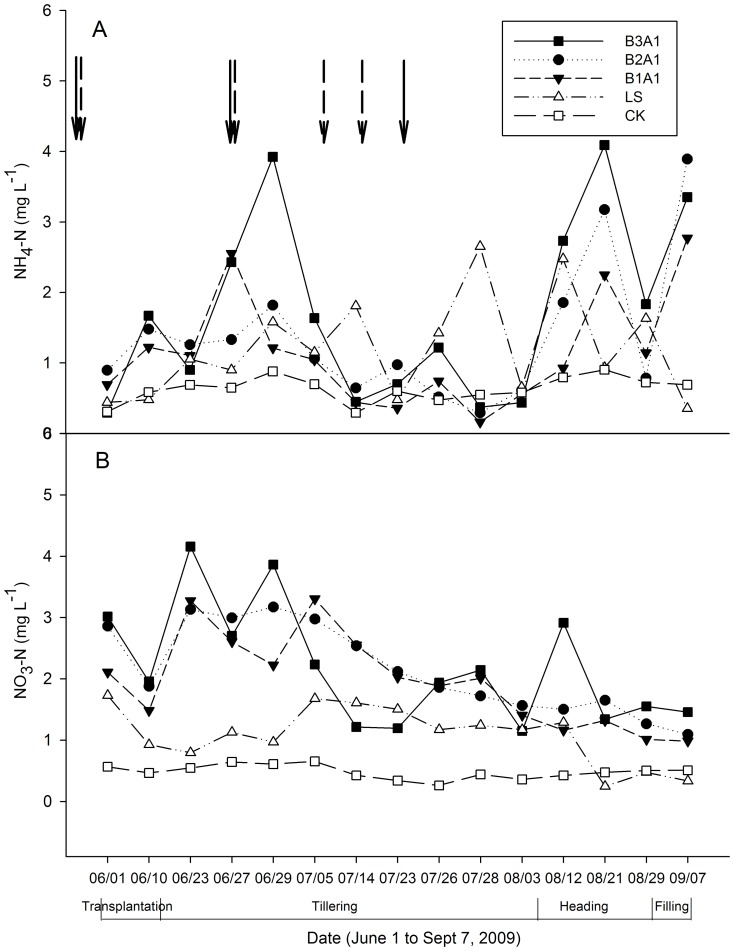
Concentrations of NH_4_-N and NO_3_-N in leachate at the 50 cm soil depth under different fertilization treatments. A: NH_4_-N; B: NO_3_-N. The solid and long-dashed arrows indicate the date of fertilizer applications for organic treatments (B1A1, B2A1, and B3A1) and for the conventional treatment (LS), respectively. Bars represent standard deviations (*n* = 3).

Leaching of NH_4_-N and NO_3_-N during the rice growing season under different fertilization treatments is shown in Fig. 3. We observed high variance in NH_4_-N concentration among three replicates of the same treatment, and this was also the case for NO_3_-N (data not shown). Two peak concentrations of NH_4_-N were observed in organic treatments, at the beginning of tillering (late June) and at heading (mid-August), and one additional NH_4_-N peak at late-tillering (late July) in conventional treatment (LS). By PROC MIXED analysis, we found over the entire growth stage of rice, application of organic fertilizers increased NH_4_-N concentration compared to no fertilization (CK, 0.63 mg L^−1^, DF = 50.9, t = 2.76); this increase was significant for B2A1 (1.37 mg L^−1^, DF = 50.9, t = 6.04) and B3A1 (1.74 mg L^−1^, DF = 50.9, t = 7.65) but not for B1A1 (1.14 mg L^−1^, DF = 50.9, t = 5.04). The concentration of NH_4_-N that percolated from LS soils was significantly higher (1.20 mg L^−1^, DF = 50.9, t = 5.29) than from CK soils, but not different from organic fields (B1A1, B2A1 and B3A1).

Organic and conventional fertilization affected NO_3_-N concentrations in percolation water (Fig. 3) differently than they affected NH_4_-N. At the 50-cm soil depth, the average NO_3_-N concentration was lowest in CK (0.48 mg L^−1^, DF = 42.2, t = 2.79) and then increased in the order of LS (1.08 mg L^−1^, DF = 42.2, t = 6.27)<B1A1 (1.96 mg L^−1^, DF = 42.2, t = 11.33)<B2A1 (2.16 mg L^−1^, DF = 42.2, t = 12.48)<B3A1 (2.19 mg L^−1^, DF = 42.2, t = 12.67). Within the percolation waters of both the CK and LS treatments, concentrations of NH_4_-N and NO_3_-N were similar, whereas in the organic treatments (B1A1, B2A1 and B3A1) average concentrations of NO_3_-N were 21–42% higher than NH_4_-N. In contrast to the two peak concentrations of NH_4_-N, NO_3_-N declined from high concentrations at early tillering until the end of the rice harvest

### Inorganic N concentrations in percolation water at 180 cm depth

**Figure 4:**
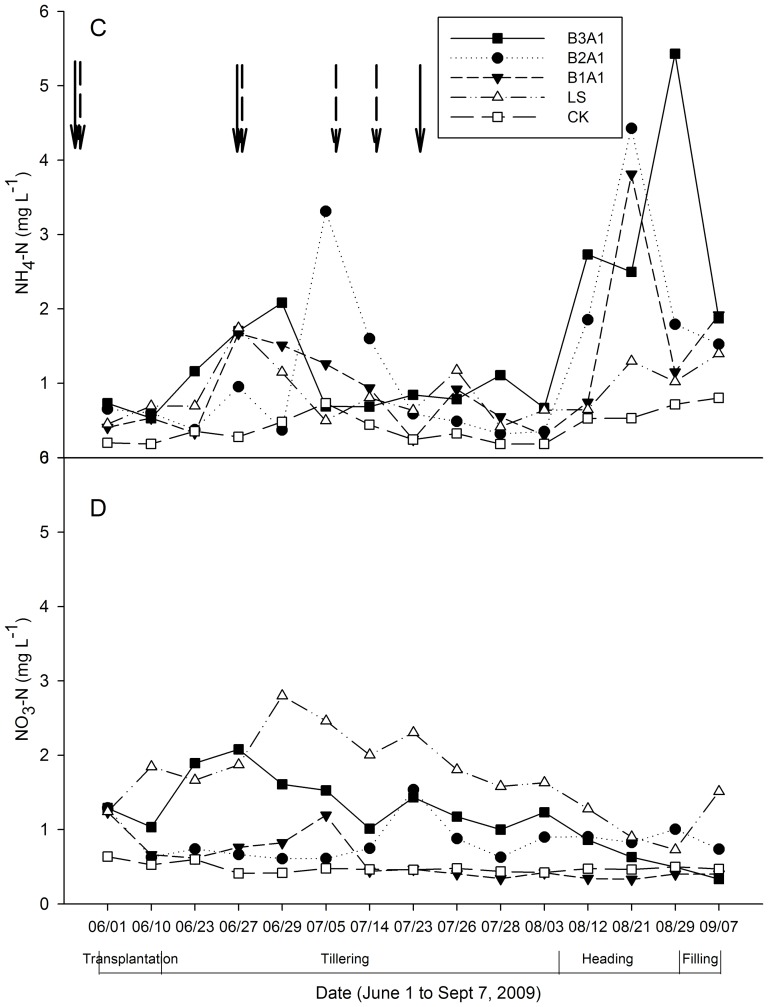
Concentrations of NH_4_-N and NO_3_-N in leachate at the 180 cm soil depth under different fertilization treatments. C: NH_4_-N; D: NO_3_-N. The solid and long-dashed arrows indicate the date of fertilizer applications for organic treatments (B1A1, B2A1, and B3A1) and for the conventional treatment (LS), respectively. Bars represent standard deviations (*n* = 3).

The average NH_4_-N concentrations at 180 cm were lowest in the CK treatment (0.41 mg L^−1^, DF = 48.8, t = 1.75) and then increased as follows: LS (0.88 mg L^−1^, DF = 48.8, t = 3.75) <B1A1 (1.08 mg L^−1^, DF = 48.8, t = 4.61) <B2A1 (1.28 mg L^−1^, DF = 48.8, t = 5.44), and B3A1 (1.57 mg L^−1^, DF = 48.8, t = 6.67, Fig. 4). These results indicated that NH_4_-N leaching increased with the increasing intensity of organic fertilization, although a high temporal variation was also observed over the monitoring period. As was found at 50 cm depth, two NH_4_-N peaks were recorded at the beginning of tillering and at the heading stage for the organic treatments, with an additional peak for LS at mid-tilling. Different from the observations at 50 cm, NH_4_ concentrations for all treatments increased from early heading, for about 1 month, until the start of paddy filling. NH_4_-N concentrations in organic rice leachates at 180 cm were similar to those at 50 cm, except for LS plots, in which values at 180 cm were significantly lower than that at 50 cm depth (0.88 vs. 1.20 mg L^−1^).

The temporal dynamics of NO_3_-N concentrations at 180 cm were similar to those at 50 cm (Fig. 4), *i.e*., as the quantity of organic fertilizer increased, leached NO_3_-N concentrations also increased. However, significantly lower concentrations of NO_3_-N were observed for organic treatments than for LS, and this was opposite to 50 cm depth where organic treatments percolated significantly higher concentrations of NO_3_-N than LS. Organic treatments had much lower NO_3_-N concentrations at 180 cm than at 50 cm (B1A1: 0.59 vs. 1.96 mg L^−1^, B2A1: 0.85 vs. 2.16 mg L^−1^ and B3A1: 1.17 vs. 2.19 mg L^−1^) while NO_3_-N concentrations in the LS treatment increased from 50 cm to 180 cm (1.08 vs. 1.70 mg L^−1^).

### Total leached inorganic N

The rice fields were flooded during the growing period from transplant (June 1) to harvest (Sep 7). At the 50 cm depth, leached inorganic N varied from 18.5 to 23.1 kg N ha^−1^ for fertilized organic treatments comparing to 12.1 kg N ha^−1^ for LS (Table 3). Of the total amount of leached inorganic N, the fertilized organic treatments showed a slight higher proportion of N as NO_3_-N (51.1∼58.9%), compared to 64.2% as NH_4_-N in LS. Higher organic fertilization rates led to higher amounts of leached NH_4_-N, but this was not the case for NO_3_-N, i.e., among the three fertilized organic treatments, the quantities of NO_3_-N remained similar. For the net total inorganic N leached, B3A1 was significantly higher than other treatments, especially compared to LS.

**Table 3:** Cumulative inorganic N leaching (kg N ha^−1^) at 50 and 180 cm soil depths for control (CK), organic (B1A1, B2A1, and B3A1) and conventional (LS) treatments during a single rice growth cycle.

Depth (cm)	Treatments	NH_4_-N (kg ha^−1^)	NO_3_-N (kg ha^−1^)	Total inorganic N leached (kg ha^−1^)	Net inorganic N leached (kg ha^−1^) a b
50	CK	3.4 c	0.0 c	3.4 d	n.a.
	LS	7.8 b	4.3 b	12.1 c	8.7 c
	B1A1	7.6 b	10.9 a	18.5 b	15.2 b
	B2A1	9.6 ab	12.6 a	22.2 a	18.8 ab
	B3A1	11.3 a	11.8 a	23.1a	19.8 a
180	CK	0.4 c	0.0 d	0.4 d	n.a
	LS	1.1 bc	1.7 a	2.7 ab	2.3 ab
	B1A1	1.4 ab	0.2 d	1.6 c	1.2 b
	B2A1	1.7 ab	0.5 c	2.2 bc	1.8 ab
	B3A1	2.0 a	1.0 b	3.0 a	2.5 a

a After subtraction of the leakage quantity measured in CK.

b Different letters in a column denote a significant difference between treatments at the 0.05 level.

At 180 cm, far less total inorganic N was percolated than at 50 cm for all treatments. For fertilized organic treatments (B1A1, B2A1 and B3A1), total inorganic N leached at 180 cm was only 9-13% of the amount leached at 50 cm; for the LS treatment, it was 26%. Relatively higher proportions of inorganic N were leached as NH_4_-N (>68%) from fertilized organic production systems, whereas 61% of leached inorganic N from LS was NO_3_-N. Among all organic treatments, B3A1 leached the highest quantity of inorganic N, significantly higher than other organic treatments, but interestingly, not higher than LS.

For the purpose of calculating N losses, we considered the inorganic N present at the depth lower than 180 cm (Table 4) to be that which could no longer be utilized by crops, and could potentially reach surface or ground water bodies. These data were used to assess the environmental performance of organic versus conventional rice production using two indicators: the amount of inorganic N leached per unit yield of rice (kg N Mg^−1^ grain) and the percentage of inorganic N leached per unit applied N (% loss) at 180 cm depth. Regarding leachate per unit yield, we found that LS and organic treatments were not significantly different, i.e., a similar quantity of inorganic N was leached per production unit yield of rice (0.21∼0.34 kg N Mg^−1^ grain). However, in terms of percent N loss, LS leached significantly more inorganic N in total N input (0.78%) than did organic production (0.32∼0.60%).

**Table 4:** Amount of inorganic N leached at 50% of N input.

Depth (cm)	Treatments	Rice yield (Mg ha^−1^)	N input (kg ha^−1^)	Net leached inorganic N per Mg of rice grains (kg N Mg^−1^) b	Proportion of net leached inorganic N in total N input (%) a b
	CK	3.6 d	n.a.		
	LS	9.4 a	297	0.93 b	2.9 bc
50	B1A1	5.6bc	193	2.69 a	7.9 a
	B2A1	6.9bc	498	2.72 a	3.8 b
	B3A1	7.6 b	802	2.62 a	2.5 c
	CK	3.6	n.a.	n.a.	n.a.
	LS	9.4	297	0.25 a	0.78 a
180	B1A1	5.6	193	0.21 a	0.60 ab
	B2A1	6.9	498	0.26 a	0.36 b
	B3A1	7.6	802	0.34 a	0.32 b

a “Net N leached” was calculated by subtracting the amount of N leached in the control treatment (CK) from total treatment values.

b Different letters in a column denote a significant difference between treatments at the 0.05 level.

## Discussion

### Inorganic N concentration in percolation water

In flooded paddy soils, aerobic and anaerobic metabolisms occur in close proximity [18], [19], [20]. At the 50 cm depth in the sandy loam soil that we studied, in organic treatments, the presence of the oxidized zone close to the reduced soil zone was conductive for the transformation of NH_4_-N mineralized from organic manure to NO_3_-N. However, the continuous anaerobic conditions (under flood irrigation, until 1 week before harvest) at the 180 cm depth maintained NH_4_-N as the main form of inorganic N, more so than NO_3_-N. Particularly, in continuously flooded paddy soils with abundant organic substrate and a limited availability of electron acceptors, the reduction of NO_3_-N to NH_4_-N would be more efficient than the formation of N_2_, so NH_4_-N concentrations can be an order of magnitude higher than those of NO_3_-N [21], [22], [23], [24]. This situation also occurs in soils with low CEC and a low content of exchangeable base cations [19], [25], [26], [27]. From the depth of 50 cm to 180 cm, average NH_4_-N and NO_3_-N concentrations decreased by about 5–10% and 47–70%, respectively, in fertilized organic treatments (B1A1, B2A1 and B3A1). The smaller decrease in NH_4_-N concentration, compared to NO_3_-N, may resulted from continuous decomposition of organic fertilizer and the limited soil adsorption capacity for NH_4_-N [19], [25], [28]. From 50 cm to 180 cm in LS soils, there was a 27% decrease in NH_4_-N, but a 57% increase in NO_3_-N, indicating that mineral fertilization may have led to an increased downward movement of NO_3_-N, compared to organic fertilizer, and the low dentrification capacity in deeper LS soils would thus have caused NO_3_-N concentration to remain high [20]. This substantial loss of inorganic via leaching NH_4_-N from continuous flooded rice fields has also been confirmed by other studies, which reported that NH_4_-N might account for up to 92% of the total inorganic N in leachate - this large risk of NH_4_-N leaching deserves more attention than the risk of NO_3_-N loss [23], [27].

During the vegetative phase of rice growth, NO_3_-N concentrations were higher than those of NH_4_-N, whereas the opposite was observed during the reproductive phase (Figs. 3 and 4). Rice requires more NH_4_-N than NO_3_-N during the vegetative stage, contributing to a suppression of NH_4_-N concentrations in leachate [29], [30]. As rice plants shifted into their reproductive stage, NH_4_-N was continuously mineralized from the organic fertilizers, but the anaerobic conditions prevented nitrification into NO_3_-N, both in the upper and lower soil profiles [23], [31]. Unlike NH_4_-N, the NO_3_-N concentration in percolation water declined from transplant until harvest mainly because of continuous uptake by the plants throughout the growing season [32]. The two peaks observed in NH_4_-N concentrations were also driven by fertilization and environmental conditions (higher temperature). At the beginning of tillering (∼June 29), air temperature was close to its annual maximum (Fig. 1), which may have promoted the mineralization of organic fertilizer. However, the observed increases in NO_3_-N concentrations lagged behind those of NH_4_-N, because urea or organic fertilizer must first be transformed to amide N, then to ammonium and further nitrified to NO_3_-N [28].

### Comparison of N utilization and loss from organic and conventional rice production

Organic and mineral fertilizers influenced rice yield differently, through differential effects on yield components such as the number of panicles per hill, number of grains per panicle, and grain weight. In our experiment, topdressing with castor bean meal in combination with increasing manure application resulted in higher grain weight, but mineral fertilizer still produced a larger number of panicles and grains per panicle [33]. A previous study revealed that increased mineral N supply to rice increases the amount of dry matter translocated into the grain [34]. However, if organic and mineral fertilizers are used together, higher numbers of panicles and grains per panicle can be achieved because of improved nutrient balance [35]. As the yield increased, more N was removed from the soil, but not in proportion with the increase in N input, such that N utilization efficiency (NUE) declined. Rice grown under conventional production showed the highest NUE (29.6%), which was significantly higher than that of organic treatments (8.7–17.6%, Table 5). This difference in NUE between conventional and organic production has been demonstrated in many other studies [8], [32], which is attributed to slow release of N from organic fertilizers that limits N uptake by crops, and also maintains the continuous loss of N via leaching or other channels [22].

**Table 5:** Nitrogen budget for rice production.

Treatments	N input from fertilizers (kg ha^−1^)	N removed by rice (kg ha^−1^)	N removed by straw (kg ha^−1^)	Total N removed by rice crop (kg ha^−1^)	N budget (kg ha^−1^)	N utilization efficiency (%) a
CK	0	40	13	53	−53	n.a
LS	297	100	41	141	156	29.6 a
B1A1	193	59	28	87	106	17.6 b
B2A1	498	85	29	114	384	12.2 c
B3A1	802	87	36	123	679	8.7 d

a Different letters in a column denote a significant difference between treatments at the 0.05 level.

Environmental performance per se, in terms of N loss through leaching is a key concern for maintaining water quality and the integrity of organic farming. When comparing organic and conventional paddy rice production, N leaching losses are a tradeoff with rice yield; for example, if high yields are to be achieved, relatively high amounts of inorganic N must be supplied and may be lost. In our study, the highest yield of organic rice (7550 kg ha^−1^) was achieved using the highest rate of N application (B3A1, 802 kg N ha^−1^; Table 4); yield in these plots amounted to 80% of that achieved through conventional treatment (LS), which received inputs of only 297 kg of N ha^−1^. Furthermore, organic production did not always perform well in terms of inorganic N leaching per unit yield of rice. In fact, if organic fertilization was intensified enough to obtain a “conventional” yield, even more N would be leached per unit yield than currently occurs in conventional production. Organic treatments with relatively higher N input (B2A1 and B3A1) tend to have lower overall rates of inorganic nitrogen loss than the LS treatment, but this came at the cost of a much lower rice yield. In the current study, the N input levels in organic treatments (except for B1A1) were higher than most conventional rice production in China, where for a single growth cycle N application varies from 200 to 300 kg N ha^−1^ [36]. In the Ili River Valley, rice production had an average N application rate of 234 kg ha^−1^, equal to the national average. This generates additional doubt that organic production can help to reduce N leaching from paddy production. In our experiments, 2.9–7.9% (8.7–19.8 kg ha^−1^) of total applied N was lost through leaching at 50 cm, and 0.32–0.78% (1.2–2.5 kg ha^−1^) was lost at 180 cm, whereas other studies involving organic fertilization reported losses of 0.1 to 15% [13], [16], [31], [37], [38]. Based on these findings, we believe that organic rice production in the Ili River Valley would not reduce (or may even increase) N leaching compared to conventional production, especially when organic fertilizer input is high enough to achieve conventional yields.

High reliance on external nutrient supply, especially from non-organic/conventional sources, has become a concern for large-scale organic production in recent years [39], [40]. Organic fertilizers are able to meet crop N requirements, but this is achieved through high application rates, which could be 1) mineralized to release a sufficient amount of N for rice growth, and 2) synchronized between N mineralization and crop uptake [10]. However, higher organic N input can cause higher N losses through leaching, as shown in our experiments. In addition, N in paddy soils can also be denitrified to the powerful greenhouse gas N_2_O [14], an additional significant environmental impact that was not measured in our study. Ju et al. [5] summarized that rice production in China using 300 kg N ha^−1^ per season contributed denitrification N losses of approximately 36%, which are markedly higher than losses from volatilization (12%) or leaching (0.5%). Here, we assume that denitrification and volatilization losses for mineral fertilizer in our study would be roughly comparable to those reported by Ju et al. [5] and Guan et al. [41]. Besides inorganic N, organic N in the leachate was not measured in our study, but it could eventually enter water bodies and cause of eutrophication. In addition, given that conventional rice has a higher NUE (29.6%), lower N input, and higher crop yield, N leaching from conventional rice production would not pose as high risk of contamination to local water systems as from organic rice production [10].

## Conclusions

This study indicates that inorganic N concentrations in leachate decreased as soil depth increased, but the decrease was significantly larger in organic than in conventional paddies. NO_3_-N tended to remain in the upper soil profile of organic paddies, whereas in conventional soils, NO_3_-N migrated further downward. In organic paddy soils, NH_4_-N accounted for a substantial portion of inorganic N in the leachate due to the organic manure decomposition and denitrification process under the continuous flooding conditions. In terms of the N leached per unit yield of rice, organic fertilization did not perform better than conventional fertilization in all cases, particularly for the organic production with higher rice yields. Consistent with this, conventional production showed higher N utilization efficiency (29.6%) compared to organic production (8.7–17.6%). We conclude that converting conventional rice production to organic production in the Ili River Valley of Central Asia will not reduce N leaching into local water systems, especially given the high-load application of organic manure to maintain high rice yields. A longer period study with integrated monitoring of N loss through leaching, volatilization, nitrification and denitrification is necessary to compare the overall performance of organic versus conventional rice production.
